# Whipping Creams: Advances in Molecular Composition and Nutritional Chemistry

**DOI:** 10.3390/molecules29245933

**Published:** 2024-12-16

**Authors:** Khadija Florence Dabo, Christine Chèné, Anne-Laure Fameau, Romdhane Karoui

**Affiliations:** 1 Adrianor, 62217 Tilloy-Lès-Mofflaines, France; f.dabo@adrianor.com (K.F.D.); c.chene@adrianor.com (C.C.); 2 University of Artois, University of Lille, University of Littoral Côte d’Opale, University of Picardie Jules Verne, University of Liège, INRAE, Junia, UMR-T 1158, BioEcoAgro, 62300 Lens, France; romdhane.karoui@univ-artois.fr; 3 CNRS, INRAE, Centrale Lille, UMET, University of Lille, 59000 Lille, France

**Keywords:** whipping cream, foam, proteins, fats, surfactant, plant-based

## Abstract

Whipping cream (WC) is an oil-in-water (O/W) emulsion used in food industry that can be transformed into aerated foam. The cream market has expanded significantly, driven by consumer demands for healthier and higher-quality products, leading to significant scientific research and innovation. This review focuses on formulation challenges related to ingredients such as fats, emulsifiers, and stabilizers, and how these components interact to form a stable emulsion and foam structure. Many studies have aimed to enhance the physicochemical, functional, and nutritional characteristics of WC by fine-tuning formulation parameters. A major focus was to address the health concerns linked to the high saturated fat content in milk fat (MF) by developing healthier alternatives. These include modifying the fat content, developing low-fat formulations, and introducing plant-based substitutes for dairy creams. The participation of additives to improve the properties of whipping cream was also investigated in many recent studies. The use of plant proteins, hydrocolloids, and emulsifiers has been explored, highlighting their effectiveness in enhancing emulsifying and foaming properties. This review summarizes recent advancements in whipping cream formulation, emphasizing the role of additives and alternative ingredients in meeting consumer preferences for healthier, more sustainable whipping cream products with enhanced functional, sensory, and nutritional properties.

## 1. Introduction

Whipping cream is an oil-in-water emulsion widely used in the food industry, as-is or transformed into an aerated foam that can have pleasant sensory characteristics and a stable foamy texture when used for desserts [1]. The whipping cream market has seen significant growth, with its value expected to rise from 819.1 million in 2021 to 1268.5 million by 2029 according to the Data Bridge Market Research [2]. This has led to an increase in scientific research on whipping creams, exploring its functional properties [3,4,5], production process [6,7], and how to improve its nutritional properties [8,9]. In recent years, many companies have offered new and innovative products to meet the demands of consumers.

Historically, the production of whipping cream was achieved through the natural skimming of milk due to the inherent difference in density between the fat globules and the aqueous phase of the milk. Subsequently, numerous innovative technologies have been developed, including those based on skimming and centrifugation, as well as those involving the assembly or reconstitution of WC with dairy ingredients [10]. Whipping cream is commonly composed of water, semi-crystalline fat, sugars, protein, stabilizers, and low-molecular-weight emulsifiers [11]. The stability of this emulsion is crucial; if too stable, foam formation is hindered, while too unstable leads to flocculation and phase separation of emulsion [12,13]. The semi-crystalline state of fat plays a significant role in the stability of the foam structure. During the process of whipping, fat droplets collide with one another, and crystals in one droplet may pierce the interfacial layer of the adjacent droplet, leading to fat coalescence [14]. Early research focused on the partial coalescence of fat, essential for the formation of a stable foam structure [15,16,17,18]. Recent studies have expanded to include the quality characteristics of whipping cream, such as its physical, functional, rheological, and nutritional properties [3,19,20,21]. Consumers demand high-quality nutrition, healthy and environmentally friendly ingredients, and excellent sensory qualities, while traders seek better storage and whipping properties to reduce costs and increase commercial value [22].

The milk fat in whipping cream contains relatively large amounts of saturated fatty acids which contribute to the high intake of these nutrients and therefore increase the risk of obesity and cardiovascular disease [22]. Thus, the high-fat content poses health risks, prompting the development of low-fat alternatives. That is why the research has intensely focused on the development of low-fat formulation, and explored the effects of various additives (hydrocolloids, proteins, emulsifiers, and lipid compounds) on WC and the final nutritional properties of whipped cream [5,23,24]. Another recent trend in the whipping cream industry has been the development of plant-based alternatives to traditional whipping cream, catering to the growing demand for vegan and dairy-free options [4,25,26]. Incorporating plant-based components in whipping cream manufacturing mitigates the impact of seasonal fluctuations on MF outcomes. This enhances the product’s functionality, nutritional value, and cost-effectiveness while optimizing quality and efficiency [27,28,29].

The use of additives is a necessity due to the complexity and instability of whipping cream; it also helps to enhance the functionality and quality of the product. Consequently, additives are instrumental in improving emulsification, foaming, sensory attributes, and rheological properties of WC. A variety of components, including hydrocolloids, proteins, lipid compounds, carbohydrates, and emulsifiers, are employed as additives, offering a range of benefits and drawbacks. For example, hydrocolloids such as xanthan gum (XG) have been demonstrated to improve foam stability and viscosity [30]. Similarly, proteins like whey protein and sodium caseinate (NaC) have been shown to enhance emulsification [31] and foam stability [32,33] under certain conditions (pH, hydrolysis, etc.). Emulsifiers, such as sucrose esters and triglycerol monostearate, improve overrun and stability by reducing interfacial tension and promoting the partial coalescence of fat globules [34]. Carbohydrates also play a crucial role in enhancing the whipping properties [4]. Sensory properties, including texture, color, and overall taste, are also critical for consumer acceptance.

Therefore, whipping cream is a popular product with a growing market, but it encounters obstacles pertaining to stability, fat content, and composition, as well as the necessity to enhance its quality and nutritional characteristics. The use of additives represents a pivotal aspect of confronting these challenges. This review examines the recent scientific advancements made over the past three years regarding the optimization of whipping cream composition and the enhancement of its functional and nutritional properties. The objective is to develop healthier and more appealing products that align with the needs of both consumers and industry.

## 2. Whipping Cream: Emulsion and Foam Characterization

### 2.1. Main Components of Whipping Cream and Their Key Roles

A whipping cream is defined as an emulsion that comprises three principal phases: the fatty phase, the aqueous phase, and the interface between the fatty and aqueous phase [35]. The aqueous phase typically consists of skimmed milk or water, while the fatty phase may include milk fat, vegetable fat, or a fat analog as an alternative. The third phase is the interface, composed of emulsifiers and/or stabilizers such as animal or vegetable proteins and low-molecular-weight surfactants (LMWS). Additionally, various other ingredients may be present in whipping cream recipes, including stabilizers (e.g., carrageenan) and sugars (e.g., lactose) [36]. Each of these ingredients plays a distinct role in the emulsion and the subsequent formation of foam.

Fat is a principal component of whipping cream due to its role in emulsification and foam stabilization [37]. The fat content in dairy whipping cream generally ranges from 30% to 40%, but in vegan or low-fat alternatives, this may be reduced to around 20% [5,23]. The fatty phase is combined with the soluble phase in the presence of a surface-active agent to facilitate the process of emulsification and to stabilize the fat droplets that are formed. The droplet size depends on the processing methods used (such as homogenization and fat maturation) and formulation factors, including fat composition and emulsifier properties [10]. The fat globule sizes are expected to be lower than 1 µm for industrial WC, since larger fat globules are known to increase the emulsion destabilization rate [38]. The fatty phase can derive from either dairy or plant sources. However, whatever the origin of the fat, the fat phase should exhibit low crystallization to facilitate the uniform formation of fat crystals during cooling and a high melting point to prevent crystallization/melting/recrystallization during storage, which could alter the functional properties [17,37]. The formation of crystals within fat droplets is a consequence of the maturation process, which involves the storage of the fat at a relatively low temperature (between 4 and 10 °C, depending on the specific recipe) for a minimum of 24 h following the production of whipping cream. The type of crystals formed is a function of multiple factors, including the origin of the fat, the type of emulsifier, and the temperature at which crystallization occurs. The formation of β or β′ crystals is preferable to α crystals for whipping cream due to their stability [39]. Hydrogenated vegetable oil or native oils with a high saturated fat content, such as coconut or palm, are commonly employed as partial or total substitutes for milk fat [29]. When used alone or in combination with other vegetable oils, they exhibit a diverse range of crystallization and melting behaviors, which can impart novel properties to the cream in terms of functionality (stability under thermal shock, ability to expand, etc.) [10].

The aqueous phase is composed of water or skimmed milk, which are commonly used to adjust the fat content of dairy whipping creams and also serve as a continuous phase in emulsions where soluble components such as proteins and sugars are solubilized prior to emulsification. The soluble phase typically constitutes approximately 50 to 60% of the total recipe.

Milk proteins are naturally present in skimmed milk or native cream, where they act as surface agents. It is possible to incorporate supplementary milk or vegetal proteins into a whipping cream recipe at a concentration ranging from 0.5 and 2% [40]. In this product category, caseins are typically the most utilized, particularly sodium caseinate, even in plant-based WC [10]. In response to the growing demand for naturality, plant proteins derived from soy, chickpeas, and kidney beans are increasingly utilized as caseinate replacers [5,41]. The proteins are expected to adsorb rapidly at the interface and reduce interfacial tension, preventing the destabilization of emulsions and maintaining the stability of air bubbles [36].

Additionally, a variety of components are incorporated into whipping creams to optimize their functional, physicochemical, rheological, and sensory characteristics. These components include emulsifiers such as lecithin and stabilizers such as carrageenan. The principal function of these additives is to strengthen the emulsion and stabilize air bubbles, thus preventing phase separation or collapse. Emulsifiers have the capacity to displace or coexist with proteins at the interface, thereby influencing the formation of interfacial layers and the properties of whipped cream [42]. Emulsifiers contribute to the stabilization of the WC emulsion by adsorbing at the oil–water interface, which induces a reduction in interfacial tension and prevents the separation of fat. In the foaming process, the emulsifiers facilitate the formation of a more robust structure surrounding the air bubbles. Proteins with a high molecular weight can partially stabilize the fats. Therefore, it is essential to utilize LMWS to develop a more robust membrane that can support long-term emulsion storage [34]. Stabilizers are expected to form a cohesive, resistant monolayer with the objective of stabilizing the emulsion and foam. Carrageenan is typically found in whipping cream, but other stabilizers, including starch, gum, and pectin, can be employed as alternatives [14]. The addition of these stabilizers serves to enhance the structural integrity of the whipped cream by thickening the soluble phase, thereby providing a firmer structure that effectively prevents foam collapse and syneresis. Ingredients such as sugars slightly enhance the flavor and can also influence the physical properties of whipped cream. They can be directly added during production and during whipping. They behave as thickening agents to improve emulsion and foam stability [14].

All these components function collectively to facilitate a stable emulsion, thereby optimizing the production of the desired fluffy texture when whipped.

### 2.2. Whipping Cream: Mechanism of Formation and Stabilization

In whipping cream, the key phenomenon is the so-called partial coalescence required to obtain a stable semi-solid foam system with a desired texture after whipping. However, this phenomenon only needs to occur during whipping and partial coalescence should be avoided during the long-term storage of the whipping cream. During the process of whipping, the collision of fat globules containing solid fat results in the formation of partially coalesced fat globules, which subsequently form a membrane that covers air bubbles and stabilizes the whipped foam [7,34]. The created fat droplets induce the formation of a 3D network of interconnected semi-solid droplets, leading to an increase in the apparent viscosity of the product. When the solid fat content is less than or equal to 15%, the phenomenon is referred to as total coalescence. If the solid fat content is between 30 and 45%, this is called partial coalescence, and a solid fat content over 50% is considered fully coalesced [38].

**Figure 1:**
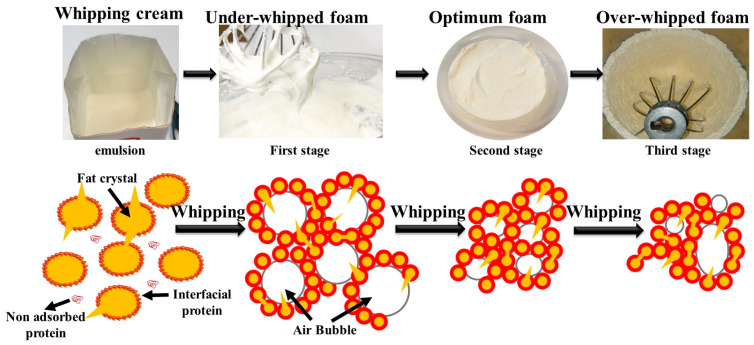
Schematic showing the mechanisms of coalescence and whipping inflation of fat globules in whipping cream depending on the whipping time.

As illustrated in Figure 1, the emulsion undergoes a three-stage transformation into foam during the whipping process, as described by Han et al. [6]. Initially, whipping causes the formation of large air bubbles within the system. Then, the protein present in the soluble phase adsorbs onto the surface of these bubbles, thereby stabilizing the initial foam. During this phase, fat droplets collide with each other, primarily due to shear-induced partial coalescence. Subsequently, under continuous shearing, these large air bubbles undergo a reduction in diameter, accompanied by an increase in the number of partially coalesced fat droplets. Ultimately, the partially coalesced fat droplet forms a network structure under the protein thin membrane that initially surrounds the bubbles, imparting stiffness to the whipped cream structure [6,14].

The protein layer plays a crucial role in maintaining the stability of emulsions and foams. It accomplishes this by wrapping around the fat droplets and air bubbles, forming a protective barrier that prevents them from merging and collapsing [6]. In commercial preparations, stabilizers such as carrageenan or xanthan gum are frequently incorporated to improve the capacity of whipping cream to retain air and sustain its structure over time [14]. It is also imperative to regulate the temperature of whipping cream during the storage period and prior to whipping. It is recommended that dairy-based WC be stored at a low temperature to maintain the integrity of the fat crystals. Prior to usage, whipping cream with dairy or plant-based ingredients should be stored at a temperature between 0 °C and 4 °C to ensure foamability. Furthermore, the duration and speed of whipping are of consequence. If the cream is over-whipped, the fat droplets will coalesce entirely, which will result in the breakdown of the foam structure and a collapse rather than a stable foam [26].

An understanding of the composition of whipping cream and the role of each ingredient allows manufacturers to tailor the properties of whipped cream and meet the needs of consumers and commercial applications. This ensures both the organoleptic and functional properties of WC.

## 3. Effect of Emulsifiers at the Interface

Fat crystallization is crucial to the formation and stabilization of whipped cream, and the variability of the crystal structures affect the partially coalesced network formed during whipping, which influences whipped creams’ stability [43]. Milk fat with appropriate melting properties and a high saturated fatty acid content is commonly used to obtain optimal functionality even if it presents health risks. Using anhydrous milk fat (AMF) is a way to decrease the possible impact of issues pertaining to fat origin (seasonality, type of cow, cow feed, etc.). However, AMF’s characteristics are influenced by various factors, making standardization challenging. Whipping cream that is non-dairy based is traditionally made using hydrogenated palm kernel oil (HPKO), which contains trans-fatty acids and saturated fats. Depending on their composition, fats have different crystallization profiles, which impact their emulsifying and foaming properties. Blending AMF with HPKO is a practical solution, as HPKO has a high melting point and a high medium and long-chain saturated fatty acid content, contributing to desirable melt-in-the-mouth characteristics [29]. The compatibility of AMF and HPKO, which both crystallize in the β′-form, makes this fat blend ideal for whipped cream. However, challenges such as flocculation and coalescence during storage necessitate the comprehensive regulation of fat crystallization and interfacial properties. Moreover, hydrogenated palm kernel oil contains trans fatty acids and saturated fats, which pose health risks. To mitigate these risks, fat substitutes using highly unsaturated liquid vegetable oils are being developed. However, substituting liquid vegetable oils for solid fats in whipped cream destabilizes its structure, leading to low firmness but increases foam stability. Emulsifiers play a crucial role in the whipping and foaming process by inducing fat globule destabilization and promoting partial coalescence. Emulsifiers can reduce protein adsorption at oil–water interfaces through the ‘orogenic displacement’ mechanism. This ability of surfactants to displace proteins varies according to their chemical structure, hydrophilic–lipophilic balance (HLB), and interactions with bulk fats. However, formulation rules are still not clear in the literature. That is why recent studies focus on the determination of the relationship between the surfactant nature, HLB, and the ability to replace proteins at the interface, facilitating the penetration of fat crystals during whipping and perhaps contributing to partial coalescence.

### 3.1. Low-Molecular-Weight Surfactants

Several recent studies have focused on the addition of lipophilic emulsifiers and their impact on the crystallization of the fat blend and the stability of the whipping cream. For example, Li et al. examined the addition of sorbitan monostearate (Span-60) with an HLB of 4.7, sucrose ester of stearate S-170 with an HLB of 1.0, lactic acid esters of monoglycerides (LACTEM) with an HLB of 4.0, sorbitan monooleate (Span-80) with an HLB 4.3, and sucrose ester of oleate O-170 with an HLB of 1.0, into a fat blend composed of AMF and HPKO in a 3:2 ratio [43]. The results showed that Span-60 and S-170, which are rich in stearic acid, exhibited strong nucleation-inducing abilities and good emulsifying properties. These emulsifiers formed tiny and uniform crystals in fat blends, resulting in small and ordered fat globules in emulsions and stable foam structures in whipped cream. LACTEM showed moderate effects due to its weaker nucleation-inducing ability. In contrast, Span-80 and O-170, which are rich in oleic acid, had poor nucleation-inducing abilities and emulsifying properties, leading to loose crystals and a decrease in whipped cream stability. The study also found that the crystallization temperature and the specific fatty acid composition of the emulsifiers significantly influenced the crystallization behavior and stability of the whipped cream. Thus, stearic acid-based emulsifiers, particularly Span-60 and S-170, are effective in enhancing the stability and sensory properties of whipped cream by promoting the formation of tiny and uniform crystals and stable foam structures. LACTEM also improves stability but to a lesser extent. Oleic acid-based emulsifiers, Span-80 and O-170 are less effective due to their poor nucleation-inducing abilities and emulsifying properties [43].

Another study used sucrose ester S370 (HLB 3, 80% sucrose diester and triester, 20% sucrose monoester, palmitic acid–stearic acid = 3:7) and the authors examined the effect on HPKO fat crystallization and whipping properties [44]. The addition of S370 had a minimal effect on droplet size, interfacial protein concentration, and the viscosity of the emulsions before whipping. However, S370, acting as a heterogeneous nucleating agent, induced nucleation, led to the formation of smaller crystals in the bulk mixture and emulsions. These smaller crystals, which could not effectively pierce the fat droplet interface, retarded fat coalescence during the whipping process. Consequently, the addition of S370 resulted in a lower degree of fat coalescence, a reduced interfacial protein concentration, a shorter optimum whipping time, and a decrease in the overrun of whipped cream. At lower S370 concentrations (<0.05 wt.%), large crystals and an inhomogeneous fat crystal network led to lower firmness and bubble stability in whipped cream samples. At 0.05 wt.% S370 concentration, a homogeneous crystal network resulted in whipped cream with high firmness and bubble stability. However, at higher S370 concentrations (>0.05 wt.%), a lower interfacial protein concentration and smaller crystals were observed, leading to a weaker aerated structure and less stable whipped cream.

The impact of different monoglycerides on the whipping abilities and partial coalescence behaviors of dairy aerated emulsions based on AMF was also studied recently focusing on three commercial monoglycerides: glyceryl monostearate (GMS), glycerol monooleate (GMO), and glycerol monolaurate (GMLa) [45]. The study found that low levels of monoglycerides (<0.25%) have minimal effects, while higher concentrations (0.50–0.75%) significantly improve the whipping performance by enhancing fat crystallization and interfacial properties. Moreover, GMS and GMLa were effective in improving the whipping abilities of aerated emulsions by enhancing fat crystallization and reducing interfacial protein concentrations. In contrast, GMO had less impact on these properties, resulting in poorer whipping performance.

**Figure 2:**
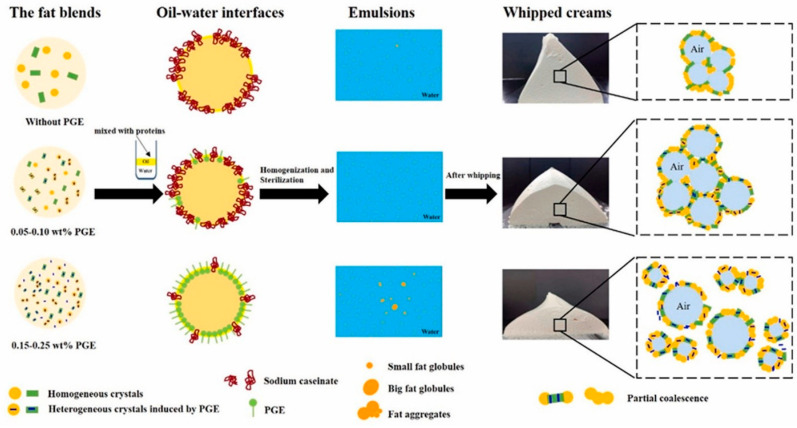
Schematic representation of the stabilization mechanisms of the oil fat droplets in the absence or presence of PGE at different concentrations, showing the replacement by PGE of sodium caseinate at the interface. The effect on the resulting foam after whipping is illustrated. Reproduced from [29]. Copyright 2023, Elsevier.

Moreover, the impact of polyglycerol ester (PGE) on the crystallization of fat blends (AMF:HPKO = 3:2) and the interfacial properties, and how these factors influence the quality of whipped cream, was recently studied (Figure 2) [29]. The PGE used was a fully esterified polyglycerol, with an average glycerol polymerization degree of 3 and a fatty acid composition of only stearic acid. The results indicate that PGE significantly influences the thermal behavior and crystal morphology of fat blends. At concentrations below 0.10 wt.%, PGE coexists with sodium caseinate at the oil–water interface, reducing interfacial tension and increasing the interfacial elasticity modulus (E′) and interfacial protein concentration, thereby enhancing emulsion stability. However, at concentrations above 0.10 wt.%, PGE displaces NaC, leading to a decreased E′ and a decrease in interfacial protein concentration, resulting in larger, irregular fat globules and emulsion instability. At low concentrations, PGE accelerates nucleation, forming small and uniform crystals, while at high concentrations, its nucleation inducing ability reach a plateau. Whipped cream properties, including partial coalescence, interfacial protein concentration, firmness, and stability, are optimized at PGE concentrations between 0.05 wt.% and 0.10 wt.%.

By comparing these studies, we can conclude that both the nature and concentration of the lipophilic emulsifier play crucial roles. Lipophilic surfactants with unsaturated chains (low melting temperature), such as GMO, Span 80, and sucrose ester O-170, are less effective at enhancing emulsifying and whipping properties due to their limited nucleation-inducing abilities, as they form a fluid layer at the interface [46]. In contrast, surfactants with stearic chains (with high melting temperature) are highly effective at improving the stability and sensory qualities of whipped cream by promoting the formation of small crystals and therefore a stable foam. These findings offer valuable insights for producing high-quality whipped cream through the addition of a controlled surfactant. However, the reader needs to keep in mind that the exact mechanism of these long-chain LMWS is still not clearly described in the literature, which could explain some of the discrepancies. Indeed, depending on the LMWS used and the process of emulsion production, fat crystallization may nucleate at two different locations: inside the oil droplets or at the surface of the droplets [46].

### 3.2. Native or Modified Dairy and Plant-Based Proteins

Proteins can fulfill diverse functions in whipping cream, acting as emulsifiers, stabilizers, viscosity-enhancers, or foaming agents. Bovine milk proteins are commonly employed in aerated dairy products to facilitate the formation of emulsions and stabilization. Caseins, which constitute approximately 80% of milk proteins, are preferred due to their conformational structure, which ensures their surface properties. For example, in the presence of LMWS, whey proteins are more prone to desorb due to their reduced flexibility (globular structure) in comparison to caseins (micellar structure) [10]. The findings of Marinova et al. indicate that casein is a more effective stabilizing agent than whey protein, forming a thicker and more resilient interfacial layer around the fat globules of WC and reducing surface tension at the oil–water interface [33]. The presence of proteins can influence several parameters of whipping creams, including the size of the fat droplets, the viscosity, the emulsion stability, and the foam stability. These effects are dependent on the concentration of proteins and their origins [10].

Whipping creams are traditionally based on dairy products. Unlike native dairy cream, recombined dairy cream (RDC) is made by combining dairy ingredients with emulsifiers and can be modified for various food applications. Bovine milk proteins, including caseins, are commonly used in aerated dairy products to facilitate the formation and stability of the emulsion. Caseins exist as micelles and can be processed into concentrate (MCC) or isolate (MCI) in their native state or modified using different technologies, including calcium caseinate (CaC) and sodium caseinate. A recent study explores how these different forms of casein affect the properties of RDC [47]. The authors investigated the impact of these three casein products (MCC, CaC, and NaC) on the stability and whipping properties of RDC, varying the concentrations of the casein products (0.5%, 1.5%, and 2.5%, *w*/*w*). The mean diameter of the MCC was found to be approximately equivalent to that of native micelles, whereas the presence of micelles was not observed in caseinates, likely due to protein disaggregation [47]. Instead, they formed large aggregates, which contributed to their relatively large particle sizes, with the CaC exhibiting the largest particle size. The highest droplet size and surface protein concentration in RDC were observed in the MCC sample, followed by CaC, and then NaC. As the protein concentration increased from 0.5% to 1.5%, the fat size decreased and the surface protein concentration increased for all samples. It was determined that an increase in casein concentration generally resulted in enhanced RDC stability attributed to the smaller particle sizes and high viscosity. However, this was not observed in the 2.5% CaC cream. Higher protein concentrations typically resulted in an increase in viscosity, with sodium caseinate creams demonstrating the highest viscosity at each concentration level. The degree of partial coalescence was found to be higher in MCC and CaC creams, which contributed to the enhancement of whipping properties. However, NaC cream was unable to be whipped into a stable aerated foam. In contrast, the MCC and CaC creams exhibited excellent whipping properties, indicating that MCC may be a more suitable ingredient for RDC preparation. The whipping properties of MCC and CaC creams were observed to be optimal at higher concentrations, whereas NaC creams were unable to form stable foams. The firmness and serum loss measurements indicated that the MCC and CaC creams at a 2.5% concentration exhibited the greatest stability against syneresis. The study’s findings indicate that MCC is the most suitable casein for RDC preparation, exhibiting superior whipping properties that could probably be attributed to the larger steric repulsions [47]. Therefore, the properties of RDC can be modified and improved using different dairy ingredients.

In consideration of the market trends, consumer perceptions regarding health and safety, and commercial considerations, recent studies have focused on plant-based whipping creams, particularly in recent years. The challenge lies in balancing the stability of the emulsion and the foaming ability required for whipping cream by using plant proteins to work in the same way as dairy proteins. Soy protein, an alternative to milk proteins, has excellent nutritional value and emulsifying properties. However, its less flexible structure limits its emulsifying functionality. The enzymatic modification of soy protein isolates (SPIs) to produce hydrolysates has been explored to increase their emulsion properties. The study by Wang et al. examined the effects of various soy proteins and their hydrolysates on the stability and texture of recombined soy-based whipping cream (RSWC) [5]. The study employed a range of soy protein types, including soy protein isolate (SPI), 7S and 11S fractions, and their hydrolysates, namely soy protein hydrolyzed by pepsin (SPHPe) and soy protein hydrolyzed by papain (SPHPa) [5]. The whipping cream emulsions were prepared by combining the fat phase (20% of coconut oil) with the water phase containing the soybean oil body (28%), different soybean proteins (4%), sucrose (13%), and deionized water (35%). Soybean oil bodies are natural sources of pre-emulsified oil. The study shows that different soy proteins significantly affect the physical characteristics and whipping properties of RSWC. The lowest apparent viscosity and shortest whipping time were observed for SPHPa, indicating that this is the most readily whipped. However, it demonstrated the poorest stability, indicating that the hydrolysates produced small polypeptides that reduced viscosity. Additionally, it exhibited the highest overrun, indicating that it incorporated the greatest amount of air during whipping. Furthermore, SPHPe demonstrated favorable overrun characteristics while exhibiting enhanced stability, rendering it a more suitable option for industrial applications. SPHPe forms a robust protein network that effectively resists foam collapse and fat destabilization, which are crucial for achieving high-quality whipped cream. The confocal micrographs indicated that the small peptides derived from hydrolyzed proteins displaced oil body proteins at the interface, resulting in the partial destabilization of fat globules and enhanced fat coalescence [5]. Hydrolyzed soy proteins, particularly SPHPe, can be effectively used in the production of an RSWC with sufficient overrun and whipping stability, offering a possible alternative to traditional dairy-based whipping creams.

To summarize, all the studies revealed that to enhance the foaming characteristics of whipped creams, it is optimal to utilize milk proteins, particularly caseins, in their native state. Conversely, when plant-based proteins are employed in their original form, due to their limited solubility, the resulting properties are observed to be inferior. In order to enhance the functionality of plant-based proteins, it is necessary to subject them to a series of modifications, including hydrolysis.

### 3.3. Surface-Active Agent Complexation

The use of only surface-active agents, such as plant-based proteins or LMWS, is typically insufficient to stabilize both WC emulsion and the subsequent foam formed, as illustrated by the aforementioned work. The incorporation of a mixture of emulsifiers or the formation of a complex such as gel-like particles or Pickering emulsion represents a viable approach to enhance the functionalities of surface-active agents in WC. A Pickering emulsion is a type of emulsion stabilized by solid particles, which adsorb at the interface of immiscible liquids (e.g., oil and water) to prevent coalescence. These particles create a physical barrier, enhancing emulsion stability.

A recent study investigated the interaction and synergetic effects of SPI and its hydrolysates with varying concentrations of monoglycerides at the air–water/oil interfaces in recombined low-fat whipping cream. The WC was formulated with 20% palm oil, 18% carbohydrate, 0.22% stabilizers, and 0.25–1.00% monoglycerides. The proteins used included native soy protein isolate (NSPI), commercial soy protein isolate (CSPI), SPHPe, SPHPa, and NaC. The results indicated that increasing the monoglyceride concentration improved the texture, whipping properties, and stability of the whipped cream by displacing the adsorbed protein from fat globules, building a firmer structure of fat aggregates, and stabilizing trapped air bubbles. SPHPa whipped cream showed a similar overrun, stability, and texture to NaC, while SPHPe led to low overrun and weakened structure due to the high proportion of β-conglycinin. SPHPa whipped cream showed similar properties to NaC for the studied formulations. The high proportion of β-subunits in SPHPe reduced the partial coalescence, resulting in a low overrun and weak structure. The study provides insights into the interaction between soy protein molecules and monoglycerides at the oil–water interface and their impact on the properties of low-fat whipped cream [5]. The results contradict the study of Fu et al. described previously [48]. We can suppose that the differences could be due to the difference in the oil phase used (coconut oil versus palm oil), the presence of monoglycerides, and/or the presence of a soybean oil body in the water phase. More work needs to be carried out on soy protein isolates and hydrolysates to better understand the differences between the studies.

**Figure 3:**
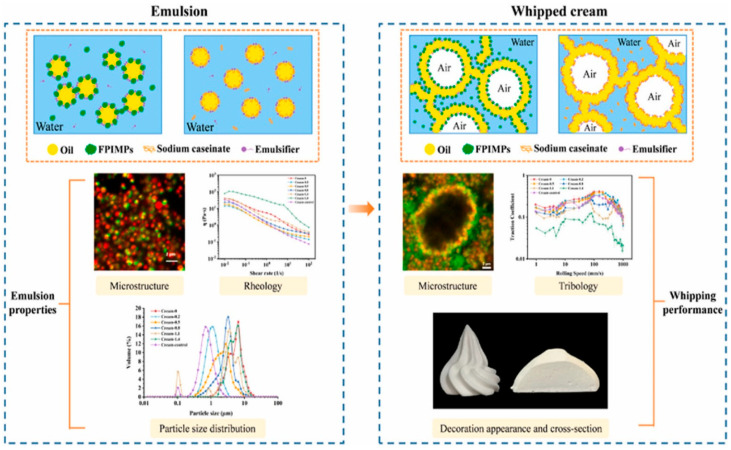
Schematic representation of the stabilization mechanisms of the oil fat droplets with sodium caseinate versus faba bean microgels and their corresponding whipping properties. Reproduced from [49]. Copyright 2024, Elsevier.

Another recent study examined the foaming properties of whipped cream stabilized by faba bean protein isolate microgel particles (FPIMPs) as a substitution for sodium caseinate (Figure 3) [49]. The faba bean protein was employed as a microgel particle to guarantee its functionality, as it has poor solubility in its native state due to its compact structure, which is a common trait among plant proteins [50]. This lack of solubility among plant proteins can negatively affect their emulsifying and foaming properties. The utilization of microgel particles presents a novel avenue for the exploitation of plant proteins in contexts in which complete protein dissolution is a prerequisite [51]. In addition to faba bean, other proteins, including pea, peanut, and soybean, were employed in the fabrication of microgels for the stabilization of emulsions, with notable success [48,52,53,54]. Microgel particles are soft solid particles that are formed via the mechanical disruption of gelled polymers through, for example, homogenization. Proteins, as a natural polymer, can be transformed into gels through a thermal cross-linking, enzymatic cross-linking, or a combination of both, and subsequently transformed into protein microgel particles. The recipe for cream emulsions consisted of 23% palm kernel stearin, 10% sugar, 3% glucose syrup, 0.1% potassium dihydrogen phosphate, 0.6% emulsifiers, and 0.1% xanthan. Additionally, the following ingredients were utilized: (0.08%) guar gum, (0.05%) sodium carboxymethyl cellulose, and FPIMPs with varying protein concentrations (0%, 0.2%, 0.5%, 0.8%, 1.1%, or 1.4%). A control sample was produced with the same recipe in which the FPIMPs were substituted with 0.8% sodium caseinate. The physical stability of the emulsions was initially elucidated. The measurement of fat globule distribution demonstrated a correlation between the average fat size and the FPIMPs content, with an observable increase in size as the FPIMPs concentration increased (Figure 3). This phenomenon can be attributed to the propensity of Pickering particles to form aggregates with a high concentration of FPIMPs, which is detrimental to emulsifying stability. The lowest fat globule size was obtained with NaC compared to other creams with FPIMPs. These results, as reported by other researchers, indicate that sodium caseinate is more effective than protein particles in preventing droplet flocculation in whipping cream [26]. It can be observed that an increase in FPIMPs results in an elevated viscosity of whipping creams. This phenomenon can be ascribed to the enlargement of fat globules [43]. However, sodium caseinate was more easily displaced from the oil–water interface than protein particles, which have a high absorption energy [55]. The measurement of foaming properties demonstrated that the creams containing NaC exhibited the most optimal foamability results. The replacement of proteins at the interface by LMWEs modified the overrun rate and whipping time. In the case of creams containing FPIMPs, the highest overrun and lowest whipping time were observed with a high FPIMPs content (0.8–1.4%) (Figure 3). With an increase in FPIMPs content, the number of protein particles in the liquid phase increases, playing a beneficial role in foam stabilization. This is achieved via the formation of a network that prevents drainage and ensures the rigidity of the foam structure [23].

### 3.4. Pickering Emulsion Formation

Another recent trend is to use particles or complexes to form Pickering emulsions, which are known for their stability due to the irreversible adsorption of particles on the interface. Zein nanoparticles (ZNP) have garnered attention due to their hydrophobic nature. Cao et al. investigated the potential of zein colloidal particles (ZCPs) to enhance the mechanical properties of whipped cream made with highly polyunsaturated oils (HPUO) [26]. The researchers found that the addition of ZCPs significantly improved the overrun and foam stability of the whipped cream during the whipping process. The ZCPs-reinforced whipped cream exhibited desirable hardness and maintained its shape better than the control samples without ZCPs. Unlike conventional whipped cream, which relies on partial coalescence of fat globules for hardness, the ZCPs-containing samples did not show partial coalescence during whipping. Instead, the particle-packed droplets in the emulsion tended to aggregate, enhancing the structure of the triphasic air–HPUO–water system. Thus, the ZCPs reinforced the whipped cream. The authors suggested that the ZCPs stabilize oil droplets by forming a rigid armor around them, which prevents partial coalescence and promotes the formation of a gel-like network. This network, along with the competitive adsorption of emulsifiers and ZCPs at the oil–water and air–water interfaces, stabilizes the air bubbles and enhances the foam’s rigidity. The aggregation of ZCPs-packed droplets further reinforces the network, contributing to the improved mechanical properties of the whipped cream. This study demonstrated that ZCPs could effectively improve the mechanical properties of HPUO-based whipped cream, making it a potential strategy for developing healthier aerated foods with non-hydrogenated and less-saturated oils.

**Figure 4:**
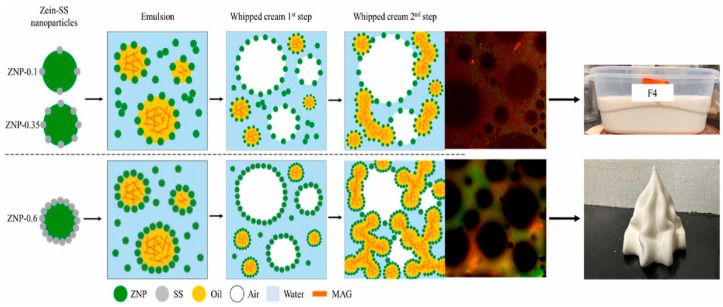
Schematic representation of the stabilization mechanisms of the oil fat droplets containing an oleogel based on monoglyceride using a complex based on zein nanoparticles (ZNP) and sodium stearate (SS). The effect of the whipping process on the resulting foam stabilization and properties is illustrated as a function of the quantity of sodium stearate used in the complex with ZNP. Reproduced from [25]. Copyright 2024, Elsevier.

Previous studies have demonstrated the successful stabilization of emulsions using zein-based nanoparticles combined with other substances like polysaccharides [56] and phenolic acids [57]. Grossi et al. developed a plant-based whipped cream by utilizing plant proteins and unsaturated oils formulated through a zein nanoparticles–sodium stearate complex (Figure 4). The complexes were combined with monoacylglyceride (MAG)-based oleogels to increase the solid fat content of vegetable oils [25]. In this study, the researchers functionalized insoluble zein with sodium stearate, significantly enhancing its solubility, emulsifying, and foaming properties. However, the use of a high amount of sodium stearate can increase potential consumer concerns. The study aimed to reduce the amount of sodium stearate by forming zein–sodium stearate nanoparticles and using oleogels as the oil phase. The hypothesis was that these nanoparticles could stabilize Pickering emulsions and incorporate air upon whipping, with the gas bubbles being stabilized by partial coalescence of the fat droplets, resulting in a stable plant-based whipped cream. A form of ZNP-SS was produced and characterized in terms of molecular interactions, wettability, and surface activity. Moreover, their ability to stabilize oil-in-water emulsions and foams in the presence or absence of MAG was evaluated. The results show that the ZNP-SS complex was stabilized through electrostatic and hydrophobic interactions. An increase in the amount of sodium stearate caused an increase in its hydrophilicity, which improved the interfacial properties of ZNP-SS. Corn oil-in-water emulsions were successfully stabilized by all the ZNP-SS, while only the ones with the highest amount of sodium stearate (ZNP-0.6) were able to create stable whipped creams due to the partially coalesced oil droplets that occurred in the presence of MAG. Thus, this study shows an innovative approach to developing stable plant-based whipped cream, representing a significant step towards creating low-saturated-fat products.

**Figure 5:**
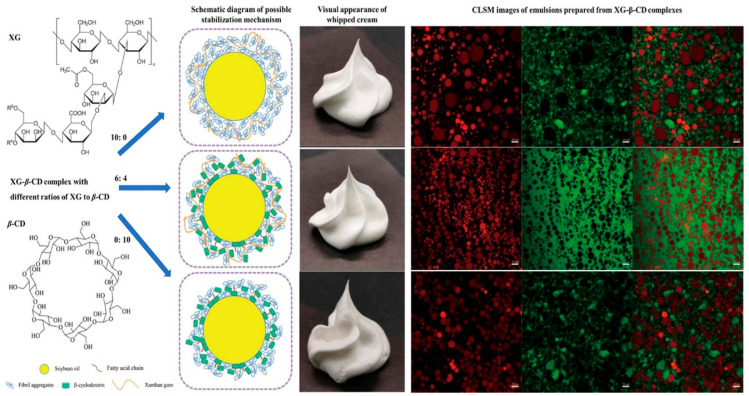
Schematic representation of the stabilization mechanisms of the oil fat droplets using a complex based on XG and β-CD depending on the ratio of the two components, the corresponding confocal laser scanning microscopy images, and photographs of the resulting whipping cream. As the concentration of β-CD in the complex increased, the droplet size of the emulsion system initially decreased and then increased, as confirmed by the average particle size measurements. At a XG–β-CD ratio of 6:4, the emulsion droplets were spherical, small in size, and uniformly distributed without any coalescence. At this ratio, red fluorescence was observed, encircling the surface of the oil droplets, forming a fluorescent ring. This indicated that the majority of XG–β-CD complex particles were tightly bound to the surface of the oil droplets, creating a spatial barrier that prevented agglomeration in the Pickering emulsions. Reproduced from [58]. Copyright 2024, Elsevier.

Another study investigated the synergistic effects of xanthan gum and β-cyclodextrin (β-CD) on the properties and stability of vegetable oil-based whipped cream stabilized by kidney bean protein aggregates (Figure 5) [58]. Xanthan gum is commonly used as a hydrocolloid stabilizer in O/W emulsions and whipped cream, contributing to long-term storage stability. It is often combined with proteins or other polysaccharides to improve emulsion stability. Xanthan gum, carrying negative charges, can interact electrostatically with positively charged macromolecules in whipped cream, enhancing viscosity and foam stability. β-Cyclodextrins, representative of oligosaccharides, have attracted attention due to their ability to enhance emulsion stability due to their amphiphilicity [59]. However, their low molecular weight limits their shaping properties in whipped cream, necessitating the auxiliary effect of large molecular polysaccharide components. The interaction between oligosaccharides and polysaccharides in composite systems, which can regulate colloid properties and improve Pickering emulsion stability, remains underexplored, particularly in vegetable oil-based whipped cream systems. The study examines the effect of β-cyclodextrin’s unique cone-like microstructure on maintaining the performance and stability of vegetable oil-based whipped cream by acting as a bridge between the hydrophilic and hydrophobic components. The materials used in the study include xanthan gum, β-cyclodextrin, kidney beans, monoglycerides and diglycerides of fatty acids, sodium stearyl lactate, corn syrup, soybean oil, and sucrose. The preparation of kidney bean protein aggregates (KPAgs) involves alkaline extraction, isoelectric precipitation, freeze-drying, and various treatments to obtain the desired protein solution. The preparation of xanthan gum and β-cyclodextrin complexes, emulsions, and whipped creams involves dissolving XG and β-CD in distilled water at various ratios, combining them with KPAgs solution, corn syrup, and sucrose, and creating a Pickering emulsion system. The emulsion is then whipped to form whipped cream (Figure 5). The study demonstrates that an appropriate XG to β-CD ratio significantly enhances the hydrogen bonding, resulting in a dense three-dimensional network structure with small, evenly distributed emulsion droplets. The unique tapered microstructure of β-CD acts as a bridge between the hydrophilic and hydrophobic components, preventing the aggregation of oil droplets and establishing a flexible support system, while XG supports the Pickering emulsion system (Figure 5). This study provides valuable insights into the production of entirely plant-based whipped cream by texturing highly unsaturated oils, addressing the issue of the inadequate intake of unsaturated oil for individuals consuming excessive animal-derived fats.

**Figure 6:**
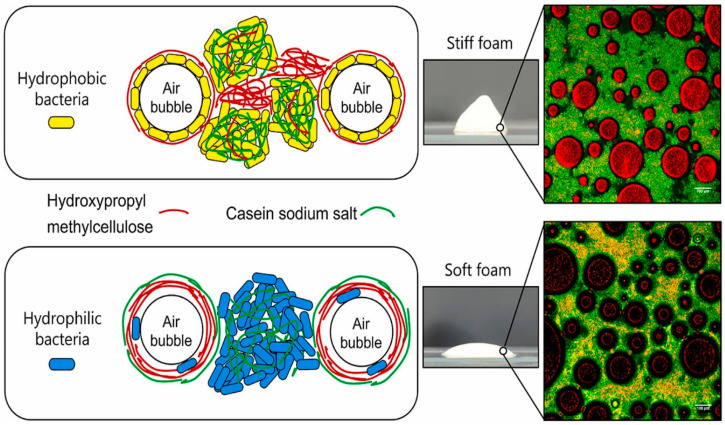
Schematic representation of the stabilization mechanisms of the air bubbles via the presence of hydrophobic or hydrophilic bacteria in the presence of hydroxypropylmethycellulose and casein sodium salt and the corresponding confocal laser scanning microscopy images of the resulting foams after whipping, as illustrated by the photographs. Reproduced from [60]. Copyright 2023, Elsevier.

A very interesting study explored the potential to use lactic acid bacteria (LAB) as structural building blocks in non-fat whipping cream analogs [60]. The research focused on replacing saturated fat content in whipping cream with LAB combined with hydroxypropyl methylcellulose and sodium caseinate salt. Microorganisms, including food-grade yeasts and non-pathogenic LAB, were reported to stabilize emulsions, double emulsions, and dry foam via a Pickering effect. The structural role of microorganisms as Pickering particles is greatly dependent on their surface chemical compositions and physicochemical properties, such as cell hydrophobicity, surface charge density, and aggregation properties. Thus, the aim of this work was to determine whether the structural functionality of solid fat globules, achieved through Pickering effects and partial coalescence, could be replicated using edible LAB with selected surface and aggregation properties. Two strains of LAB, *Lactobacillus delbrueckii subs. lactis* ATCC 4797 (LBD) and *Lactobacillus crispatus* DSM20584 (LBC), with different surface properties, were combined with NaC and HPMC to create a series of whipping cream-like suspensions. The study found that the overrun of the whipped cream was mainly attributed to the use of HPMC and NaC rather than the bacteria due to the faster adsorption of molecular surfactants compared to micron-sized bacterial cells. However, the drainage stability was systematically dependent on the strain that was used, with LBD samples allowing for nearly twice the drainage of the LBC samples. The viscoelasticity of the whipped cream was measured, showing that all samples exhibited higher storage modulus (G′) values than loss modulus (G″) values, indicating a more solid than liquid character. Formulations with LBC showed much higher G′ values than LBD samples, suggesting stronger bacterial aggregation and more solid-like properties. The aggregation of LBC was primarily enhanced by the NaC component, while for LBD samples, HPMC was more capable of promoting bacterial aggregation (Figure 6). The microstructure of the whipped cream was investigated using confocal laser scanning microscopy, revealing that the hydrophobic LBC exhibited almost full coverage at the air–water interface, indicating a stronger Pickering capability than the hydrophilic LBD. The viability of bacteria in the whipped cream was quantified, showing that neither the added components nor the whipping process had detrimental effects on bacterial viability. In conclusion, non-fat whipping cream analogs with a high overrun and drainage stability were formulated using LAB as natural structural building blocks combined with NaC and HPMC components. The more hydrophobic LBC strain created whipped cream with a higher stiffness and liquid retention ability than the hydrophilic LBD strain due to the strong Pickering stabilizing capacity and network-forming ability of LBC bacteria. The aggregation of the two strains was enhanced to differing extents by the added components, with NaC better promoting the aggregation of LBC and HPMC inducing a stronger aggregation of LBD (Figure 6). The study opens the possibility of using natural LAB as both structural building blocks and probiotic food components.

## 4. Effect of Stabilizers in the Continuous Phase

Reducing the fat content in whipped cream often results in weaker textural properties, such as reductions in firmness, viscosity, overrun, and stability against drainage. To address these issues, the addition of different stabilizers has been explored, such as fibers and hydrocolloids, to improve the texture of low-fat whipped cream. Various fat replacers, including modified whey protein isolate, rice starch, different seed gums, and glucose, have also been used to enhance the textural properties of low-fat whipped cream. Not only does low-fat whipped cream need to be achieved, but also the low sugar content WC what increase the complexity of the formulation. Therefore, a current research focus is developing whipped cream analogs with low-fat content or exploring new additives with the desired properties. The stabilization of whipped cream is a critical technical problem that has attracted much attention. Exploring the interactions between food components and finding new food additives prepared from food-grade materials are promising solutions to this problem in the food industry.

### 4.1. Effect of Sugar Addition

The study by Zeng et al. investigates the impact of glucose and corn syrup on the physical characteristics and whipping properties of vegetable-fat-based whipped creams [4]. This study analyzes how glucose and corn syrup affect the physical and foaming properties of vegetable-fat-based whipped creams. The research focuses on glucose and corn syrup and how varying concentrations of these sugars affect the emulsions’ interfacial protein concentration and apparent viscosity, as well as the overall stability and firmness of the whipped creams. It was observed that corn syrup, which contains maltodextrin, resulted in an increase in interfacial protein concentration and a reduction the coalescence of oil droplets compared to glucose. Maltodextrin protects the absorbed proteins during freezing and slows the formation of a continuous network during whipping, resulting in reduced fat coalescence and a lower firmness in the whipped cream. Despite these differences, whipped creams with corn syrup still exhibit relatively good stability, especially at higher sugar concentrations. The addition of sugars, particularly glucose and corn syrup, influences the whipping properties of the cream. Higher sugar concentrations lead to shorter optimum whipping times, an increase in fat coalescence droplets, and increased firmness and stability in the whipped creams. These effects are attributed to the sugars’ ability to increase the interfacial protein concentration and the apparent viscosity of the emulsions. In terms of practical applications, the study suggests that the partial replacement of glucose with corn syrup or maltodextrin could improve the stability and foaming properties of WC. This finding is significant for the development of low sugar whipped cream products, which are increasingly in demand due to health concerns related to high sugar intake.

### 4.2. Effect of Polysaccharides Addition

A recent study demonstrated how cellulose extracted from sugarcane bagasse using chemicals before undergoing a specific electrohydrodynamic (EHD) treatment could be a good stabilizer agent for whipping cream [61]. Cellulose, a biopolymer composed of repeating β-d-glucopyranose units linked via 1→4 glycosidic linkage, is the most abundant organic polymer and an inexpensive source of food additives [62]. Due to its highly crystalline nature, cellulose is often modified to improve its techno-functional properties such as its water absorption capacity, viscosity, and gelling ability [63]. Modified cellulose has been used as a fat replacer in various dairy products, including dairy beverages, yogurt, ice cream, and whipped cream. However, the literature on the application of cellulose modified using electrical field treatment in food products is limited. EHD treatment is a high-voltage, non-thermal food processing method that generates corona discharge, creating an electric wind that induces mass transfer and microstructural changes in biological materials. EHD treatment has been used for food-drying, nutrient encapsulation, and the extraction of food compounds [64]. The cellulose extracted from sugarcane bagasse and treated with EHD showed a partial transformation from a microcrystalline to amorphous state, enhancing its water absorption capacity and viscosity. The authors compared the addition of modified and non-modified cellulose to whipped cream with varying fat concentrations. The results showed that an increase in cellulose and fat concentrations improved the viscosity, firmness, overrun, and stability against syneresis of the whipped cream. Whipped cream samples containing modified cellulose exhibited superior textural and color qualities compared to those with non-modified cellulose. The sample with 1.5% modified cellulose and 30% fat showed preferable stability, texture, and color properties. Thus, EHD-treated cellulose fibers enhanced the stability, texture, and color of whipped cream, making it a viable option for the production of lower-fat dairy products.

A recent research article investigated the impact of basil seed gum (BSG), a novel polysaccharide, known for its thickening and emulsifying properties [65]. The authors compared the effects of BSG and κ-carrageenan on the rheological, textural, and structural properties of whipping cream [65]. The results indicate that whipping cream containing BSG exhibits a higher viscosity compared to whipping cream with κ-carrageenan. BSG significantly improved the cream’s viscosity, demonstrating a strong capacity to enhance the rigidity of the emulsion. A rheological analysis showed that BSG improves the low-frequency dependence of the elastic modulus, indicating a more rigid structure. The study also found that BSG can form a pseudo-gel network by adsorbing protein segments at the oil–water interface, creating a stronger molecular network in the whipping cream. In contrast, whipping cream with κ-carrageenan showed some degree of flocculation, likely due to non-adsorbed polysaccharides or proteins. A microstructural analysis using scanning electron microscopy revealed that whipping cream with BSG contained more voids and pores, which decrease with an increase in fat content, enhancing the foam structure. The study concludes that synergistic interactions between proteins and polysaccharides, such as BSG and κ-carrageenan, promote the development of a cross-linked network, improving the emulsifying properties of WC. However, further research is needed to explore the mechanisms behind BSG’s interactions at the oil–water interface and its impact on the microstructure of whipped cream.

A first study investigating the potential functions of hydroxypropyl methylcellulose (HPMC) in whipping cream was conducted, finding that HPMC displaced proteins from the interface and improved the textural and foaming properties of whipped cream [66]. Then, a second study investigated the emulsifying and foaming properties of mixing polysaccharide dispersions, specifically focusing on the effect of the insoluble soybean fiber (ISF) and HPMC ratio [67]. ISF is the main component of okara, obtained in large quantities during the production of soy-based products, and might provide great valorization opportunities for both the application of agricultural byproducts and the exploration of novel food additives [67]. The study explored how the interactions between ISF and HPMC influence the processing and characteristics of food systems such as whipped creams. In this study, HPMC was partially replaced by ISF, and O/W emulsions and whipped cream were prepared. The mixture of ISF/HPMC exhibited a good foaming performance after whipping, and the foaming performance was enhanced as the proportion of ISF increased. In the O/W emulsion system, the overall stability, including the acid and salt resistance, was maintained, while the heat stability and centrifugal stability of the O/W emulsions first increased and then decreased as the proportion of ISF increased. This indicated that the partial replacement of HPMC with ISF could help to improve the emulsion stability. In the whipping cream system with an increase in the proportion of ISF, the overrun, foam stability, and cream stability first increased and then decreased. The size of the fat bubbles decreased, and the elastic modulus first increased and then decreased. This indicated that the addition of an appropriate amount of ISF strengthened the network structure of whipping cream and enhanced the stability of foam and cream, thus showing its excellent application potential in whipping cream. Thus, ISF could be used to partially replace HPMC to prepare O/W emulsions and whipped cream.

As described above, hydrocolloids are efficient ingredients to improve the stability of whipping creams, but little information is available on their impact on the nutritional properties and lipid digestion. A recent study focused on the use of carrageenan and pectin in whipping cream and explored their potential to not only improve the textural properties of whipped cream, but also to retard lipid digestion [22]. Indeed, hydrocolloids can induce the flocculation of lipid droplets in the gastrointestinal tract, thereby decreasing the lipid digestion rate. The hydrocolloid solutions of carrageenan and pectin were prepared by dispersing the hydrocolloids in distilled water, followed by stirring and refrigerating for complete hydration. Fresh cream with a fat content of approximately 40% was adjusted to a 30% fat content by adding raw skimmed milk. The hydrocolloid solutions were then added to the cream, which was pasteurized, homogenized, and refrigerated to promote fat crystallization. The whipping cream was then aerated using a stand mixer, and the samples were examined for their in vitro lipid digestion properties [22]. The study found that the addition of hydrocolloids reduced the average volume–surface diameter of the lipid droplets and increased the net negative charges, indicating improved homogenization efficiency. Both pectin and carrageenan enhanced the foaming properties of the whipped cream, with pectin generally exhibiting higher overrun values. The addition of hydrocolloids also significantly reduced serum loss, indicating an improvement in foam stability. In terms of lipid digestion, both hydrocolloids caused the flocculation and/or coalescence of droplets when exposed to pH changes during the digestion model, resulting in creaming and phase separation of the emulsions. Carrageenan was found to be more effective in reducing the rate of lipid digestion, as evidenced by the reduced amount of released free fatty acids compared to pectin. In conclusion, the study demonstrates that hydrocolloids like pectin and carrageenan can be used in whipping creams to both improve foaming properties and retard lipid digestion. These findings suggest that the rate and extent of lipid digestion can be controlled by adding certain types of polysaccharides, which may be useful for developing healthier whipped cream products with better functional properties.

### 4.3. Effect of Protein–Polysaccharide Complex

In the literature, several examples have been provided of the use of polysaccharides in combination with other food ingredients to improve the properties of whipping creams. For example, protein–polysaccharide complexes, due to their high emulsifying ability and suitable surface activity, have been considered as potential fat-replacers in low-fat whipping cream [68]. These complexes can improve the stability of oil–water emulsions by forming new additives with various interfacial properties. The combination of proteins and polysaccharides yields an emulsion with enhanced functional properties, such as improved stabilization and emulsification. For example, the study by Rezvani et al. demonstrated the effects of a protein–polysaccharide complex (sodium caseinate, carboxymethyl cellulose, and locust bean gum) on the physical and textural characteristics of low-fat whipping cream [68]. The authors used the response surface methodology to optimize the proportions of protein and polysaccharides to find the best qualitative properties including overrun, apparent viscosity, firmness, average particle size, drainage, and color parameters. Their results showed that the proposed combination of stabilizers improved the physical and sensory characteristics of low-fat whipped cream, enhancing its apparent viscosity, firmness, average particle size, and overrun while reducing drainage. A sensory evaluation revealed that the most desirable treatment optimization for low-fat cream contained 0.35% sodium caseinate, 0.15% carboxymethyl cellulose, and 0.15% locust bean gum.

Another study focused on the effect of a protein–k-carrageenan mixture [24]. A low-fat whipping cream was formulated by substituting the removed fat content with a blend containing water, SPI, and k-carrageenan gum. SPI are excellent emulsifiers that can prevent oil droplets from coalescing, while k-carrageenan can increase viscosity and reduce serum phase leakage, enhancing WC stability. The influence of SPI and k-carrageenan content on the stability of the WC was examined using a simplex–centroid design to evaluate the optimum formulation. Various properties, such as foaming, viscosity, textural properties, and emulsion stability, were determined. The physical stability of the whipped cream was evaluated by measuring overrun, viscosity, adhesiveness, and firmness. The results showed that water proportion significantly affected foamability, with a higher water content leading to an increase in foam capacity. SPI also increased foam volume, while high levels of k-carrageenan decreased overrun and increased viscosity. The stability of the foam was ensured by the presence of SPI and k-carrageenan, which provided rigidity and visco-elasticity to the films, stabilizing air bubbles. The viscosity of the samples varied, with higher values being observed in formulations with more k-carrageenan. A texture analysis showed that a higher k-carrageenan content resulted in a harder and more stable foam. Emulsion stability was maintained by the presence of emulsifiers, k-carrageenan, and SPI, which prevented phase separation. A microscopic examination revealed differences in the microstructure of the cream samples, with monodispersed emulsions showing higher viscosity. A sensory evaluation indicated that the addition of SPI and k-carrageenan improved the sensory attributes of low-fat whipping creams. The optimum fat substitute blend contained 44.5% water, 46.4% SPI, and 9% k-CG, resulting in a product with 31.5% fewer calories than full-fat WC. These two studies highlighted the possible use of a protein–polysaccharide complex for the development of low-fat, stable, and acceptable whipping cream products.

## 5. Conclusions and Future Directions

Whipping cream is a widely used product in the food industry, and its market has grown substantially in recent years due to increasing consumer demand for healthier and higher-quality options. This growing interest has driven substantial scientific research and innovation. In this review, we highlight the most recent literature (from the past three years) focusing on the effects of proteins, low-molecular-weight surfactants, and stabilizers in whipping cream formulations. In the future, a major focus will be on creating healthier alternatives, such as whipping creams fortified with probiotics and formulations with reduced levels of saturated fats and sugars. The development of low-fat, low-sugar, and functional whipping creams is a key priority and requires further attention. Consumers also continue to seek more “natural” foods and “clean label” products without synthetic additives, meaning that even if functional and nutritional improvements are achieved through additives, the health impact and perceived naturalness of these additives must be carefully considered. Another important trend is the growing development of plant-based substitutes for dairy creams. However, more research is needed to match the sensory and functional properties of traditional dairy creams. Ongoing studies into plant proteins (isolates, solid particles, microgels, etc.) are essential to create alternatives that replicate the texture, stability, and overall quality of dairy-based whipped creams. All the studies confirmed that to enhance the functionality of plant-based proteins, it is necessary to subject them to a series of modifications, including hydrolysis, particle formation, or microgels, to create a gel-like network or to use them in synergy with other emulsifiers. Furthermore, a better understanding of the role of LMWS is crucial for producing high-quality whipping cream through controlled surfactant use, as their exact mechanisms are still not fully described in the current literature. Clarifying the nucleation process—whether it begins inside the oil droplets or on their surface—will also be key to controlling the final properties of whipped creams.

In summary, the development of whipping creams with improved nutritional and functional properties, as well as non-dairy alternatives, presents both significant opportunities and challenges for future research.

## Data Availability

Not applicable.
